# Meetings of Societies

**Published:** 1948

**Authors:** 


### Meetings of Societies

### Bristol Medico-Chirurgical Society

THE sixth meeting of the Society was held on Wednesday, March 11th. Dr. R. C. Clarke and Dr. J. Naish gave accounts of **"The Changing Incidence of Disease."**

The seventh meeting was held on April 14th, when Mr. J. J. Mason-Brown came from Edinburgh to describe his **" Experiences in the Treatment of Injuries to Blood Vessels."**

The eighth meeting was held on May 12th and devoted to three short papers : Professor Rendle Short —**" Thyroidectomy or Thiouracil "** [p. 48] ; Dr. H. J. Gibson, **" The Morbid Histology of Rheumatoid Arthritis,"** beautifully illustrated by lantern slides; and Dr. R. H. Norman, **Cerebral Diplegia following Birth Injury "** [p. 43].

